# Synaptic Plasticity Linked to Ionotropic Glutamate Receptors After Nicotine Exposure

**DOI:** 10.2174/011570159X365159250311142852

**Published:** 2025-04-07

**Authors:** Aqsa Kazmi, Eun Sang Choe

**Affiliations:** 1 Department of Biological Sciences, Pusan National University, 63-2 Busandaehak-ro, Geomjeong-gu, Busan 46241, Republic of Korea

**Keywords:** Actin, cytoskeleton, dendritic spines, glutamate, nicotine, synaptic remodeling

## Abstract

Tobacco dependence is a chronic, relapsing disorder with significant socioeconomic and health impacts that lead to considerable morbidity and mortality worldwide. Nicotine is the primary component responsible for the initiation and continuation of tobacco use. Nicotine exposure causes multiple alterations in the structure and function of the brain’s reward system. Evidence shows that synaptic plasticity, a key event that modifies neural circuit structure and function, is largely influenced by changes in glutamatergic neurotransmission in the forebrain’s reward pathways. It is now widely accepted that α-amino-3-hydroxy-5-methyl-4-isoxazolepropionic acid receptors (AMPARs) modify synaptic strength within the reward circuitry. Dendritic spines, the primary sites of synaptic plasticity, exhibit an array of structural adaptations in size and shape influenced by neural activity, which correlates with alterations in the strength of synaptic connections. Such alterations in dendritic spine morphology largely depend on the remodeling of the underlying actin cytoskeleton. The dynamics of the actin cytoskeleton are regulated by several modulators, including actin-binding proteins, protein kinases, and small GTPases. This review focuses on the restructuring of the dendritic spine machinery and the relevant changes in synaptic strength mediated by AMPARs in key brain areas involved in addiction. However, our understanding of the neural pathways governing the emergence and significance of the structural and functional changes that lead to addiction-like behaviors after prolonged nicotine exposure remains insufficient. Comprehending these essential neural processes could deepen our insight into the progression and maintenance of nicotine dependence.

## 1 INTRODUCTION

Drugs of abuse act on cortico-limbic reward circuits, which include the prefrontal cortex (PFC), the striatum, and the limbic system, including the hippocampus and amygdala [1]. Among these reward circuits, the striatum serves as a key structure that integrates glutamatergic inputs from cortical, thalamic, and limbic regions to encode drug-related stimuli [2, 3]. The striatum consists of the caudate and putamen (CPu), which regulate decision-making and habit formation, and the nucleus accumbens (NAc), which serves as a crucial center for neuronal circuits that process motivation and reward related to drug addiction [4-8].

The NAc receives extensive glutamatergic afferents from the PFC [9], amygdala [10], hippocampus [11], and thalamus [12]. The CPu receives glutamatergic inputs from the motor cortex [13, 14]. Each of these glutamatergic projections to the striatal subregions is believed to control various aspects of behaviors related to drug addiction [15-18]. Studies have indicated that nicotine enhances glutamatergic neurotransmission in the brain's reward circuits by stimulating various nicotinic acetylcholine receptors (nAChRs), which are predominantly expressed on the presynaptic terminals of glutamatergic neurons [19, 20]. These neurochemical alterations are associated with behavioral changes after long-term exposure to nicotine [21, 22]. For instance, nicotine binding to α7 nAChRs in the dorsal striatum of the rats increases behavioral sensitization [23, 24]. These findings emphasize the role of glutamatergic neurotransmission in mediating nicotine-induced drug reward and addiction.

Over 95% of neurons in the striatum are medium spiny neurons (MSNs) that primarily consist of γ-aminobutyric acid (GABA) neurons [25]. Glutamatergic inputs converge onto the heads of dendritic spines in the GABAergic MSNs of the striatum. The neurotransmitter glutamate, released at glutamatergic synapses, binds to ionotropic glutamate receptors (iGluRs), including *N*-methyl-*d*-aspartate (NMDA), α-amino-3-hydroxy-5-methyl-4-isoxazolepropionic acid (AMPA), and kainate receptors [26]. Among the several iGluRs, AMPA receptors (AMPARs) play a central role in mediating the majority of fast excitatory neurotransmission and synaptic plasticity [27-29].

Frequent small protrusions present on the dendrites of MSNs, known as dendritic spines, receive the most excitatory synaptic inputs in the brain. It is well recognized that activity-driven modifications in synaptic efficacy modulate spine morphology, influenced by changes in the actin cytoskeleton [30]. Actin serves as the primary component of the cytoskeleton in dendritic spines and has been thoroughly studied as a determinant of synaptic plasticity related to changes in spine morphology [31]. Actin-dependent changes in the cytoskeleton play a significant role in mediating structural and functional modifications of synaptic plasticity in dendritic spines [32]. Studies have shown that drug dependence is associated with modifications in both the structure and function of synapses within the reward circuitry [33, 34].

This review focuses on the activity-dependent structural plasticity in dendritic spines linked to AMPARs associated with nicotine addiction. Starting with actin and its dynamic control by actin-binding proteins (ABPs), such as cofilin, we explore the modifications in the postsynaptic AMPARs, including AMPAR trafficking in dendritic spines. In addition, we discuss some neuromodulators downstream of AMPARs that mediate intracellular signaling events regulating actin dynamics and dendritic spine morphology. These include several protein kinases, such as calcium/calmodulin-dependent protein kinase II (CaMKII), p21-activated kinases (PAKs), LIM domain kinase (LIMK), and small Ras homology guanosine triphosphatases (Rho GTPases). The regulation of intracellular signaling events associated with actin dynamics could provide insight into the possible mechanisms underlying nicotine-induced alterations in spine morphology and behavioral responses.

## 2 DYNAMIC ROLE OF THE ACTIN CYTOSKELETON IN NICOTINE-INDUCED PLASTICITY OF DENDRITIC SPINES

The dendrites possess a framework called the cytoskeleton, which is responsible for shaping and structuring spines. Actin serves as the primary structural element of the cytoskeleton, and its organization and dynamics are involved in modulating and sustaining the morphology and physiology of dendritic spines [35, 36]. Actin is a monomer of the globular protein G-actin that forms F-actin filaments after polymerization [37]. The two forms of actin (F-actin and G-actin) go through a cycle of polymerization and depolymerization called actin cycling. The remodeling of the actin cytoskeleton is carried out by actin cycling *via* a balance between the assembly and disassembly of actin filaments, which alters the shape and size of the spines [38]. The transition between the polymerization and depolymerization of F-actin and G-actin during actin cycling is strictly regulated by an abundance of signaling, scaffolding, and ABPs [39, 40].

In dendritic spines, the actin cytoskeleton not only maintains the structural integrity but also acts as an anchor for several postsynaptic proteins, including AMPARs present in postsynaptic density (PSD), which are involved in activity-dependent alterations in synaptic efficacy [41]. Thus, the actin cytoskeleton may contribute to the maintenance of synaptic plasticity through multiple pathways. One potential pathway involves the control of actin dynamics, which can modify the shape and structure of dendritic spines. Another involves the postsynaptic modification and trafficking of AMPARs, influencing changes in synaptic efficacy. Therefore, actin plays a pivotal role in the structural and functional plasticity of dendritic spines.

### 2.1 Regulation of Actin Dynamics by ABPs

Actin cytoskeleton reorganization and its interaction with ABPs and their regulatory molecules mediate the structural and functional changes in dendritic spines [42, 43]. A variety of ABPs, such as cofilin, Wiskott-Aldrich syndrome protein (WASP), the WASP-family verprolin-homologous protein (WAVE protein) complex, and actin-related protein 2/3 (Arp2/3) complex, influence the dynamics of the actin cytoskeleton *via* several mechanisms including polymerization, depolymerization, cross-linking, branching, nucleation, and AMPAR trafficking [44]. The limitations of this review prevent an in-depth exploration of all ABPs; consequently, we concentrate on a few, notably, cofilin.

Cofilin, belonging to the actin-depolymerizing factor (ADF) family, is an actin-modulating protein that regulates the reorganization of the cytoskeleton in dendritic spines through actin cycling [45, 46].

Cofilin-mediated actin cycling is essential for the correct structure and function of synapses on dendritic spines [47]. Moreover, cofilin activity is involved in the actin-dependent growth and shrinkage of dendritic spines during synaptic plasticity. For instance, functional loss of cofilin causes abnormal spine enlargement, whereas its overactivation increases actin depolymerization, leading to immature spine formation or spine shrinkage [48]. Cofilin binding to actin is controlled by the phosphorylation of LIMK1, which renders it inactive, and dephosphorylation (or activation) by calcineurin at position 3 of a conserved serine residue (Ser 3) [49, 50]. This phosphorylation and dephosphorylation cycle of cofilin plays an important role in the reorganization of the actin cytoskeletal associated with synaptic plasticity.

Cofilin not only modifies the shape of dendritic spines but also regulates the movement and concentration of AMPARs within synapses. Studies have shown that the dynamic process of activation and inactivation of cofilin in the dendritic spine is involved in dendritic spine remodeling and AMPAR trafficking during long-term potentiation (LTP) of synaptic activity [51, 52]. A previous study revealed that phosphorylation of cofilin resulted in an elevation of AMPAR trafficking and upregulation of dendritic spine size during LTP. In contrast, long-term depression (LTD) of synaptic activity-induced dephosphorylation of cofilin led to a calcium-dependent downregulation of spine size in the dendritic spines of the rat hippocampus [53]. Another study described that during LTP, the translocation of cofilin to the postsynaptic membrane is associated with the enlargement of dendritic spines in the rat hippocampus [54]. Such findings indicate that cofilin activity is vital for mediating structural and functional alterations in the synaptic plasticity of dendritic spines [55].

Several studies showed that drugs of abuse affect neuronal functions and drug-induced behaviors by altering synaptic structure through mechanisms involving cytoskeleton reorganization mediated by ABPs, including cofilin [56-58]. For instance, withdrawal after repeated exposure to cocaine increases actin cycling in the NAc of rats, which may be responsible for the reinstatement of cocaine-seeking behavior [59]. In similar studies, cocaine withdrawal after daily administration of the drug increased the head diameter of spines in the MSNs of the NAc in rats, linking altered spine morphology to behavioral plasticity [60, 61]. Chronic nicotine exposure may influence the mechanisms of synaptic plasticity, including actin dynamics, but information exploring the effects of nicotine on the actin cytoskeleton of dendritic spines is limited. Early evidence has shown that repeated nicotine exposure alters the expression levels of actin in the cortex and hippocampus of mice, indicating drug-induced changes in cytoskeletal proteins [62]. The mechanisms by which AMPARs are linked to such changes in synaptic remodeling remain unknown.

### 2.2 Modifications in Postsynaptic AMPARs

AMPARs consist of four subunits (GluA1-4) that are highly homologous and differ mostly in the C-terminus domain (CTD) [63, 64]. In various brain areas related to addiction, most AMPARs exist as GluA1/GluA2 subunits, whereas others are GluA2/GluA3 subunits [65, 66]. Differences in subunit composition determine their binding to various proteins during AMPAR trafficking and synaptic plasticity [67].

GluA1 is an extensively studied subunit of AMPARs concerning synaptic plasticity [68, 69]. The CTD of the GluA1 subunit contains multiple sites for post-translational modifications that are fundamental for the regulation of AMPAR trafficking to and within the postsynaptic membrane [70, 71]. The CTD of GluA1 serves as a substrate for several kinases including cAMP-dependent protein kinase A (PKA) (GluA1-Ser 845), protein kinase C (PKC) (GluA1-Ser 831; GluA2-Ser 863), cGMP-dependent protein kinase G (PKG) (GluA1-Ser 845), CaMKII (GluA1-Ser 831/Ser 567), and p21-activated protein kinase 3 (PAK3) (GluA1-Ser 863) [72-75].

Studies have shown that psychostimulants affect the phosphorylation state of the GluA1 subunit by various kinases, mediating drug-induced synaptic plasticity and changes in behavior [76, 77]. For instance, repeated cocaine administration results in PKC-mediated phosphorylation of GluA1 at Ser 845 of AMPARs in the CPu of rats [78]. Similarly, an increase in phosphorylation of GluA1 at Ser 831 by PKG is associated with behavioral changes after repeated cocaine exposure in the NAc of rats [79]. A recent finding demonstrated that an increase in CaMKII-induced phosphorylation of GluA1 at Ser 831 in the dendritic spines of the MSNs of the CPu is required for the sensitization of reward-related behaviors in rats following repeated nicotine exposure [80].

Evidence suggests that phosphorylation of the GluA1 subunits at Ser 831 and Ser 845 regulates actin-dependent AMPAR trafficking, which is associated with LTP and synaptic plasticity [81]. A previous study revealed that the phosphorylation of GluA1 at Ser 863 by PAK3 results in the elevation of subunit trafficking to the postsynaptic membrane of hippocampal dendritic spines [74]. Together, these studies indicate that GluA1 subunit-specific modifications may regulate activity-induced synaptic plasticity in the dendritic spines. However, the exact actin-dependent mechanisms underlying subunit trafficking after drug exposure remain to be elucidated.

### 2.3 Actin-dependent Trafficking of AMPARs

The postsynaptic membrane of dendritic spines is enriched with AMPARs that are highly dynamic and move in and out of synapses in a manner depending on neuronal activity [82]. AMPAR trafficking includes endosomal recycling, endocytosis, and exocytosis, all of which are regulated by the actin cytoskeleton [83, 84].

A significant body of research has demonstrated that numerous actin-associated proteins, such as postsynaptic density 95 (PSD-95), synapse-associated protein 102 (SAP-102), and protein 4.1N, affect the insertion of GluA1subunit of AMPARs into the postsynaptic membrane, thereby regulating AMPAR trafficking [85-87]. A recent study has shown that during LTP, the phosphorylation of actin-associated protein 4.1N by CaMKII increases the recruitment of GluA1 subunits of AMPARs to the postsynaptic membrane. This finding reveals that actin is responsible for the trafficking of AMPARs, while protein 4.1N serves as an anchoring protein that links GluA1 with the actin cytoskeleton [88]. Furthermore, AMPAR auxiliary subunits and subunit-specific protein binding partners are also involved in the mechanisms that determine single-channel conductance properties, synapse anchoring, and AMPAR trafficking associated with synaptic plasticity and LTP [89-91]. The incorporation of AMPARs into the postsynaptic membrane plays a central role in enhancing synaptic strength during LTP [92, 93]. Collectively, these observations suggest that the actin cytoskeleton may serve as a fundamental basis for the assembly of AMPARs and their dynamic synaptic expression in dendritic spines.

Psychostimulants, such as cocaine, influence AMPAR trafficking, thereby mediating synaptic plasticity and dendritic spine remodeling. For instance, withdrawal from repeated cocaine exposure increases the surface expression of AMPARs in the dendritic spines of the NAc, along with increased spine density [94]. The majority of AMPARs in the NAc consist of GluA2-containing subunits that are impermeable to calcium [95]. Chronic cocaine exposure and withdrawal lead to an increase in the expression of calcium-permeable AMPARs (CP-AMPARs) that primarily consist of GluA1-containing homomers, lacking the GluA2 subunit in the MSNs of the NAc [96, 97]. The recruitment of GluA2-lacking AMPARs to the postsynaptic membrane increases channel conductance properties and modulates synaptic plasticity. Studies have shown that repeated cocaine exposure and subsequent withdrawal generate silent synapses in the MSNs of the NAc, which are responsible for the consolidation of synaptic transmission and cocaine-induced behavioral changes in rats [98, 99]. Actin-associated proteins PSD-95, PSD-93, and SAP-102 seem to regulate AMPAR-mediated formation and maturation of silent synapses in the NAc of mice after long-term cocaine exposure and withdrawal [100]. Chronic nicotine exposure may affect AMPAR trafficking; however, the pathways linking the dynamic regulation of actin cytoskeleton to AMPAR expression and accumulation at the postsynaptic membrane after prolonged nicotine exposure remain to be explored. Further investigation into the relationship between AMPARs and the cytoskeleton proteins they interact with after exposure to nicotine may illuminate the potential mechanisms underlying nicotine addiction.

## 3 MOLECULAR MECHANISMS OF CHANGES IN DENDRITIC SPINES LINKED TO AMPARs

Since reorganization of the actin cytoskeleton is associated with changes in spine structure and function, it is crucial to understand the signaling events that link actin cycling to synaptic plasticity [101]. Many signaling molecules connect actin and ABPs to synaptic activity and participate in the signaling pathways associated with dendritic spine remodeling. Among these regulatory molecules, CaMKII and its downstream effectors, such as the Rho GTPases and PAKs, have been extensively studied in the context of synaptic plasticity and drug abuse.

### 3.1 CaMKII

Calcium signaling results in the formation of calcium/calmodulin (CaM) complexes. This complex triggers the activation of calcium/CaM-dependent enzymes, including CaMKII, and protein phosphatases, such as calcineurin (CaN) [102]. CaMKII, activated by protein kinases, subsequently regulates several downstream pathways, leading to the remodeling of the actin cytoskeleton in the spines.

The significant abundance of CaMKII in dendritic spines indicates that it may not only have a catalytic function but also play a role in the structural reorganization of the actin cytoskeleton within these spines. Under basal conditions of neuronal activity, CaMKII bundles F-actin and maintains the stability of the shape and structure of the spines [103]. Upon activation, CaMKII undergoes autophosphorylation and releases itself from actin filaments to interact with other signaling or scaffolding molecules. This provides temporary flexibility to the actin cytoskeleton, facilitating the remodeling of the structure of dendritic spines. These findings reveal a clear mechanism by which structural dynamics of CaMKII enable a link between calcium signaling and the morphological adaptations of dendritic spines [104].

Several pieces of evidence suggest that CaMKII serves as a biochemical processor for calcium signaling during the induction of LTP and LTD [105-108]. Therefore, CaMKII has a dual function in synaptic plasticity, serving as a structural element under the basal states and as a signaling molecule during phases of LTP in neuronal activity [109]. Thus, CaMKII relays calcium signals to the dendritic spines *via* its kinase activity, thereby altering synaptic strength and structural plasticity [110, 111].

A previous study indicated that CaMKII-induced phosphorylation of GluA1-Ser 831 is involved in AMPAR trafficking in mouse hippocampal neurons [112]. Moreover, AMPAR-associated changes are involved in mediating synaptic plasticity after exposure to psychostimulants [76]. For example, the level of activated CaMKII increases alongside the upregulation of synaptic AMPARs surface expression after cocaine withdrawal in the NAc of rats, which may influence drug-seeking behavior [113]. Therefore, CaMKII may be a key signaling molecule involved in cocaine-induced synaptic plasticity *via* increased expression of synaptic AMPARs in the MSNs of the NAc.

Consistent with this, a previous study has revealed that CaMKII activity in the NAc of mice mediates nicotine-induced reward behaviors [114]. Another study described that continued exposure to nicotine can lead to increased phosphorylation of Ser 831 at the C-terminus of GluA1 subunits in the CPu of rats, which has been linked to behavioral sensitization to nicotine [80]. Furthermore, CaMKII triggers alternative pathways that regulate F-actin dynamics and dendritic spine remodeling *via* its kinase activity. The Rho family of small GTPases serves as the primary convergence point for these CaMKII-induced signaling cascades [115].

### 3.2 Small GTPases

A key regulator of the spine cytoskeleton is the Rho family of GTPases, which belong to the Ras superfamily [116]. This family includes cell division cycle 42 (Cdc42), Ras-related C3 botulinum toxin substrate (Rac), and Ras homology (Rho), and their roles in cytoskeleton-mediated dendrite morphogenesis have been well studied [117]. Generally, the activation of Cdc42 and Rac promotes actin polymerization, increasing the number of spines, whereas Rho activation inhibits actin polymerization and causes spine loss and shrinkage [118].

Several studies have revealed that the regulation of small GTPase proteins is essential for the reorganization of the cytoskeleton during structural and functional changes in dendritic spines [119]. Moreover, it is evident that the activation of Rac1 promotes the polymerization of F-actin and enhances the stability of dendritic spines. This process is mediated by the stimulation of several downstream effector proteins such as PAKs, LIMKs, and cofilin [120]. Psychostimulants, such as cocaine, activate Rac1 to mediate structural and behavioral plasticity in the CPu of rats [121]. GTPase activity is controlled bidirectionally by GTPase-activating proteins (GAPs) and guanine-nucleotide-exchange factors (GEFs). GAPs suppress GTPase activity, whereas GEFs trigger it [122]. Accumulating evidence indicates that the Rho GTPases, GEFs, and GAPs play significant roles in synaptic plasticity associated with substance abuse [123]. How these molecules regulate actin cytoskeleton dynamics during nicotine addiction remains to be investigated.

### 3.3 PAKs

PAKs are a group of serine-threonine kinases that function as downstream effectors of small GTPases, Cdc42, and Rac1. This kinase family is divided into two subgroups. Subgroup I consists of PAK1-3, whereas subgroup II consists of PAK4-6. These subgroups exhibit distinct functions, and their regulation occurs through diverse mechanisms [124]. PAKs regulate cytoskeletal dynamics mainly by the phosphorylation of various substrates. The primary downstream effector of Cdc42 and Rac1 is PAK1, the activity of which promotes the maturation of dendritic spines in hippocampal and cortical neurons [125, 126]. PAK1, *via* the regulation of LIMK1 and cofilin, mediates changes in the actin cytoskeleton that are crucial for LTP in the mice hippocampus [127]. Moreover, the knockdown of PAK3 results in the loss of mature synapses and the formation of abnormally long dendritic spines devoid of synapses in rat hippocampal spines [128]. Evidence suggests that PAK3-induced phosphorylation of GluA1-Ser 863 increases the expression of AMPARs in the postsynaptic membrane of hippocampal dendritic spines [73]. However, the exact cellular mechanism is not yet understood.

A previous study has shown that the overexpression of GEF Kalirin-7 (Kal-7) results in an increase in spine density in the NAc, as well as an increase in locomotor sensitization after chronic cocaine exposure in mice [129]. Other studies have described that the Kal-7 mediated Rac1/PAK/LIMK pathway promotes changes in cofilin activity and induces AMPAR-associated structural adaptations in dendritic spines of the NAc, along with behavioral plasticity after long-term cocaine exposure in the rodents [94, 130]. Given the importance of Rho GTPases (Rac1 and Cdc42), their upstream regulators (CaMKII and Kal-7), and downstream effectors (PAKs and LIMKs), these molecules may play a role in the nicotine-induced changes in altered cytoskeletal dynamics, particularly related to AMPAR trafficking and dendritic spine morphology.

## 4 DYNAMIC RELATIONSHIP BETWEEN STRUCTURAL AND FUNCTIONAL CHANGES IN DENDRITIC SPINES

Dendritic spines are highly plastic, and changes in their morphology are structurally correlated with alterations in synaptic strength [131, 132]. Despite their distinct and complementary roles in neuronal plasticity, there is a clear connection between the physiology of synaptic neurotransmission and the structure of the dendritic spines [133]. How neuronal activity modifies spine morphology and how these modifications affect synaptic neurotransmission are intriguing issues.

Spines display a range of structural and functional adaptations owing to changes in their input activities. The level of synaptic neurotransmission can be enhanced by either LTP or LTD of synaptic activity [134]. Numerous studies have demonstrated that LTP induces modifications in spine morphology, including changes in the size of the spine head [135-138]. Correspondingly, the intensity of LTP induction correlates with an increase in spine density, which is attributed to greater F-actin content, enhanced stabilization, and increased cofilin accumulation [139, 140]. These findings provide a link between LTP and structural changes in spines that are regulated by actin dynamics. Conversely, the induction of LTD has been reported to result in an increased loss of mature spines, a decrease in synapse number, and shrinkage of the spine head in the rat hippocampus [141-143]. Moreover, the increased diameter of the spine head results from enhanced actin turnover and AMPAR trafficking to the cell surface, leading to an increase in synaptic strength in the hippocampal neurons [144].

Drugs of abuse have been shown to alter dendritic spine morphology and subsequent synaptic plasticity [145]. For instance, long-term cocaine abuse alters spine density in the MSNs of the NAc and glutamatergic projection neurons in addiction-related brain regions in rats [130]. Studies have shown that nicotine exposure increases the dendritic spine length and spine density in the NAc and cortex of rats [146-148]. A previous study indicated that acute and long-term exposure to nicotine decreased the minimum threshold for LTP induction in the dorsal hippocampus [149]. Nicotine also regulates LTP in a manner that depends on neuronal activity in the rat hippocampus [150]. According to another study, exposure to nicotine causes lateral expansion of the spine head through direct modulation of glutamatergic synapses, which results in dendritic spine remodeling in hippocampal neurons [151]. The exact correlation between the structural and functional plasticity of dendritic spines and nicotine use remains unclear. To elucidate the underlying mechanisms, researchers aim to discern how neuronal activity affects spine morphology and how different shapes influence synaptic functions, especially during prolonged nicotine exposure.

## 5 BEHAVIORAL IMPLICATIONS OF NICOTINE-INDUCED DENDRITIC SPINE REMODELING

Several pieces of evidence suggest that changes in glutamatergic neurotransmission play crucial roles in both synaptic plasticity and drug-seeking behavior following psychostimulant abuse. For instance, long-term cocaine exposure alters glutamatergic neurotransmission in the NAc, which modulates drug-related behaviors [152]. Similarly, an increase in AMPAR-induced glutamatergic plasticity in the MSNs of the NAc is linked to behavioral sensitization and the reinstatement of cocaine-seeking behavior following repeated cocaine exposure and withdrawal in rats [153, 154]. In addition, cocaine re-exposure following withdrawal is associated with an increase in actin cycling and spine head diameter in the NAc core, leading to locomotor sensitization in rats [61].

The impact of nicotine, particularly during early withdrawal and when actively sought after during prolonged abstinence, is crucial for maintaining tobacco smoking habits. Few studies have focused on neuronal actin dynamics to investigate their influence on nicotine-induced changes in behaviors, such as the reinstatement of nicotine-seeking and relapse. A previous study indicated that nicotine-seeking behavior could be reinstated following a period of self-administration and extinction of drug-reinforced behavior in rats [155]. Another study has shown that chronic nicotine exposure is associated with an increase in branch number and length of dendritic spines in MSNs of the NAc shell in adolescent rats [156]. These observations attribute structural changes in spine morphology to repeated nicotine exposure.

Moreover, repeated nicotine exposure influences glutamatergic neurotransmission in the brain’s reward areas. A study revealed that nicotine withdrawal increases the expression of the AMPAR GluA1 subunit, suggesting that the plasticity of glutamatergic neurotransmission in the NAc is involved in the reinstatement of nicotine-seeking behavior [157]. Additionally, cue-induced nicotine-seeking is associated with the dysregulation of glutamate homeostasis within the cortico-striatal circuitry [158, 159]. These findings highlight the importance of targeting the glutamate system, including AMPARs, for nicotine-induced plasticity in the reward circuits of the brain [160].

Similarly, chronic exposure to nicotine induces structural changes in striatal circuits, such as dendritic arborization, and an increase in spine density in brain regions linked to goal-directed and habitual behaviors. These adaptations may contribute to the development of compulsive drug-seeking behavior and an increased susceptibility to relapse [161]. In brief, it has been observed that nicotine-seeking behavior after prolonged self-administration followed by withdrawal is associated with an increase in the volume density of both symmetric and asymmetric synapses, along with an increase in the length of asymmetric synapses on dendritic spines in the NAc of rats [162]. Table **1** summarizes the effects of various doses of nicotine and cocaine on addiction-related brain areas, the corresponding dendritic spine alterations, and the associated behavioral outcomes.

The exact neurobiological mechanisms underlying the incubation of nicotine-seeking are not well known. However, this phenomenon may be attributed to prolonged alterations in the structure of dendritic spines and synaptic neurotransmission in various cortico-limbic brain areas, including the amygdala [163, 164]. Events underlying the remodeling of the dendritic spine linked to the stimulation of AMPARs are postulated in Fig. (**1**). The dearth of research on synaptic plasticity occurring during the reinstatement of nicotine-seeking presents a promising opportunity in the field of nicotine addiction. By delving into this area, we can significantly deepen our understanding of nicotine-seeking and relapse behaviors that arise from tobacco consumption.

## CONCLUSION

Substance dependence arises as a behavioral outcome stemming from intricate neurological adaptations spanning various domains. We aim to provide a relatively comprehensive review of some structural and functional adaptive changes at glutamatergic synapses, primarily in the striatum, that have clear implications for addiction-related behaviors. As glutamatergic synapses on dendritic spines serve as critical sites for excitatory neurotransmission, it is essential to investigate how modifications in the structure and function of spines at the circuit level alter synaptic activity triggered by psychostimulants, particularly nicotine. Further research is required to explore the exact mechanisms underlying the regulation of actin dynamics and associated postsynaptic modifications and trafficking of AMPARs after repeated nicotine abuse. Moreover, there remains a significant gap in our understanding of the influence of nicotine on signaling pathways that lead to changes in synaptic strength and structure in the dendritic spines of glutamatergic synapses. Progress is needed to elucidate the essential events that link functional and structural plasticity and how these forms of synaptic plasticity influence nicotine addiction-related behaviors, such as withdrawal, reinstatement of nicotine seeking, and relapse. This could further contribute to our understanding of the development and persistence of nicotine dependence, potentially leading to new perspectives for creating novel and more precise pharmacotherapies for nicotine addiction.

## Figures and Tables

**Fig. (1):**
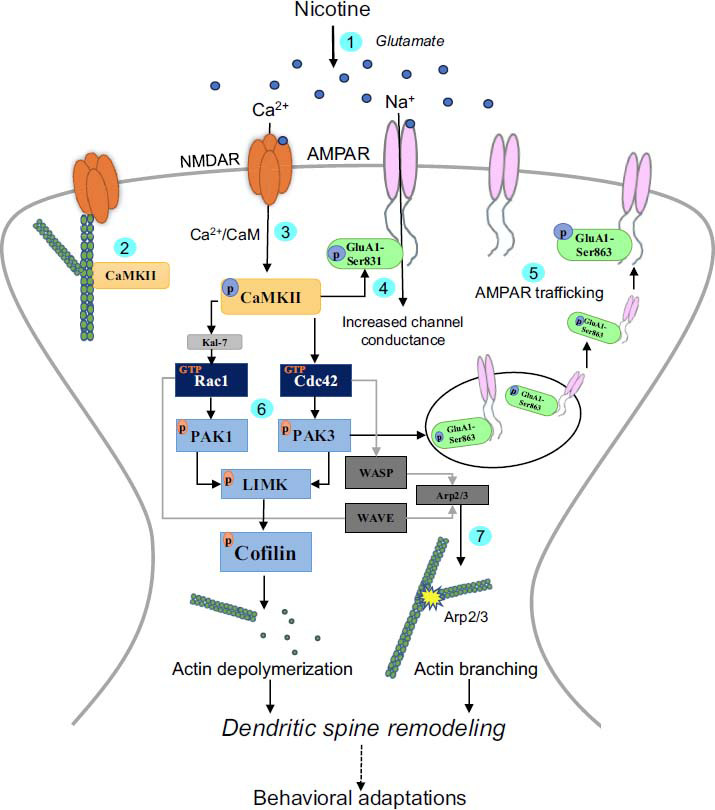
Schematic illustration of the predicted events underlying dendritic spine remodeling linked to ionotropic glutamate receptors in response to nicotine. (**1**) Nicotine exposure causes the release of glutamate from presynaptic glutamatergic terminals. (**2**) Under basal conditions, F-actin is bundled by CaMKII, maintaining the shape and structure of the spine head. (**3**) Glutamate binding to NMDA receptors (NMDARs) leads to increased calcium conductance through NMDARs, activating CaMKII. (**4**) Phosphorylation of GluA1-Ser 831 by activated CaMKII may enhance channel conductance and synaptic neurotransmission. (**5**) PAK3-induced phosphorylation of GluA1-Ser 863 results in increased trafficking of AMPARs to postsynaptic membrane. (**6**) CaMKII-mediated activation of (Kal-7-mediated) Rac1/PAK/LIMK or Cdc42/PAK/LIMK signaling cascades leads to cofilin-induced actin depolymerization and dendritic spine remodeling. (**7**) Activation of Arp 2/3 complex by the WASP/WAVE complex is involved in actin nucleation and branching, leading to the formation of new actin filaments.

**Table 1:** The effects of drugs of abuse on changes in dendritic spines, and behaviors in addiction-related brain areas.

**Drugs**	**Brain Areas**	**Animals/Dose**	**Dendritic Spine Changes**	**Behavioral Effects**	**References**
Nicotine	NAc, cingulate cortex	Rats/0.7 mg/kg	Increase in dendritic spine length and spine density	Increased locomotor activity	[146]
Nicotine	Motor cortex	Rats/0.3 mg/kg	Increased dendritic arborization	Increased motor skills	[147]
Nicotine	NAc	Rats/0.2 mg/kg	Increased number of dendritic spine branches	-	[156]
Nicotine	CPu	Rats/0.25 mg/kg	-	Increased locomotor activity	[21]
Cocaine	NAc	Rats/15-30 mg/kg	Changes in spine head diameter and spine density	-	[60]
Cocaine	NAc	Mice/20 mg/kg	Increased spine density	Changes in locomotor activity	[129]
Cocaine	CPu	Mice/20 mg/kg	Increased dendritic branching and spine density	Changes in locomotor sensitization and conditioned place preference	[121]

## LIST OF ABBREVIATIONS

AMPARs: α-amino-3-hydroxy-5-methyl-4-isoxazole-propionic Acid Receptors
CPu: Caudate and Putamen
MSNs: Medium Spiny Neurons
NAc: Nucleus Accumbens
nAChRs: Nicotinic Acetylcholine Receptors
PFC: Prefrontal Cortex
